# Postpartum fertility behaviours and contraceptive use among women in rural Ghana

**DOI:** 10.1186/s40834-018-0066-9

**Published:** 2018-08-20

**Authors:** Sebastian Kofi Eliason, Ansumana Sandy Bockarie, Cecilia Eliason

**Affiliations:** 1 Department of Community Medicine, School of Medical Sciences, University of Cape Coast, Cape Coast, Ghana; 2 FWACN School of Nursing and Midwifery, University of Ghana, Accra, Ghana

**Keywords:** Postpartum, Contraceptive use, Breastfeeding, Amenorrhoea, Sexual abstinence

## Abstract

**Background:**

Although most women would want to wait for more than two years before having another baby, their fertility behaviours during the first year following birth may decrease or increase the length of the birth interval. The objectives of this study were to: assess how protected postpartum women in the Mfantseman municipal were against pregnancy, based on their patterns of amenorrhoea and sexual abstinence; determine the timing of postpartum contraception in relation to amenorrhoea and sexual abstinence; and determine the predictors of postpartum contraceptive use.

**Methods:**

This was a prospective study carried out in the Mfantseman Municipality of the Central region of Ghana. Out of 1914 women attending antenatal clinic in the municipal within the study period, 1350 agreed to be part of the study to ascertain their postpartum fertility and contraceptive behaviours a year following delivery. These women were traced to their communities using telephone and house numbers provided and only 1003 of the women were finally traced and interviewed. The women were asked about their breastfeeding behaviour, postpartum sexual abstinence, duration of amenorrhoea and postpartum contraceptive use.

**Results:**

The mean age of the respondents was 29.9 ± 6.5 years; adolescents constituted the least proportion (3.3%) of the women. More than half (54.1%) of the women had Middle, Junior secondary school or Junior high school education. Most (43.3%) of the women were married by means of traditional rites and more than half (51.4%) of them were petty traders. The mean durations of breastfeeding, amenorrhoea and sexual abstinence were 6.6 ± 2.8 months, 7.8 ± 3.8 months and 4.4 ± 3.1 months respectively, whilst mean time of first contraceptive uptake was 3.5 ± 2.7 months postpartum. The time to first use of modern contraceptive method during the postpartum period indicates that about 50% of the women had started use of modern contraceptive methods by 2.7 months postpartum, and occured 0.6 and 3.6 months before sexual relations and resumption of menses respectively. Occupation (likelihood ratio *p* = 0.013), area of residence (likelihood ratio *p* = 0.004), mode of delivery (likelihood ratio *p* < 0.001), breastfeeding (*p* = 0.024), period since delivery (*p* < 0.001), preferred number of children (*p* < 0.001) and parity (*p* < 0.001) were found to be predictors of postpartum contraceptive use.

**Conclusion:**

Postpartum women in the Mfantseman municipal who did not use contraceptives or delayed in the use of contraceptives after birth were least likely to be protected against pregnancy in the post partum period, whilst those who adopted postpartum family planning were likely to be better protected because they were likely to adopt it within the first three months after birth and before the onset of sexual relations and first menses. The predictors of postpartum contraceptive use were breastfeeding pattern, occupation, parity, preferred number of children, period since delivery, place of residence and mode of delivery.

## Plain English text

Although most women would want to wait for more than two years before having another baby, their fertility behaviours during the first year after birth may decrease or increase the length of the birth interval. The objectives of this study were to: assess how protected postpartum women were against pregnancy, based on their patterns of amenorrhoea and sexual abstinence; determine the timing of postpartum contraception in relation to amenorrhoea and sexual abstinence; and determine the predictors of postpartum contraceptive use.

This was a prospective study carried out in the Mfantseman Municipality of the Central region of Ghana. Out of 1914 women attending antenatal clinics in the municipal within the study period, 1350 agreed to be part of the study to ascertain their postpartum fertility and contraceptive behaviours a year following delivery. These women were traced to their communities using telephone and house numbers provided and only 1003 of the women were finally traced and interviewed. The women were asked about their breastfeeding behaviour, postpartum sexual abstinence, duration of amenorrhoea and postpartum contraceptive use.

The mean age of the respondents was 29.9 ± 6.5 years; adolescents constituted the least proportion (3.3%) of the women. More than half (54.1%) of the women had Middle, Junior secondary school or Junior high school education. Most (43.3%) of the women were married by means of traditional rites and more than half (51.4%) of them were petty traders. The mean durations of breastfeeding, amenorrhoea and sexual abstinence were 6.6 ± 2.8 months, 7.8 ± 3.8 months and 4.4 ± 3.1 months respectively, whilst the mean time of first contraceptive uptake was 3.5 ± 2.7 months postpartum. The time to first use of modern contraceptive method during the postpartum period indicates that about 50% of the women had started use of modern contraceptive methods by 2.7 months postpartum, and occured at 0.6 and 3.6 months before sexual relations and resumption of menses respectively. Occupation (likelihood ratio *p* = 0.013), area of residence (likelihood ratio *p* = 0.004), mode of delivery (likelihood ratio *p* < 0.001), breastfeeding (*p* = 0.024), period since delivery (*p* < 0.001), preferred number of children (*p* < 0.001) and parity (*p* < 0.001) were found to be predictors of postpartum contraceptive use.

Postpartum women in the Mfantseman municipal who did not use contraceptives or delayed in the use of contraceptives after birth were least likely to be protected against pregnancy in the postpartum period, whilst those who adopted postpartum family planning were likely to be better protected because they were likely to adopt it within the first three months after birth and before the onset of sexual relations and first menses. The predictors of postpartum contraceptive use were breastfeeding pattern, occupation, parity, preferred number of children, period since delivery, place of residence and mode of delivery.

## Background

The 2006 World Health Report proposed a 2–3 year birth interval and a six month conception interval following miscarriage or abortion, because they ensured good maternal and child health outcomes [1]. Evidence exists that if couples could space their pregnancies by at least two years, up to 35% of maternal deaths and up to 13% of child mortalities could be averted [2–4], whilst 25% of under – five mortalities could be averted if birth intervals were at least three years [3].

Although most women would want to wait for more than two years before having another baby, their fertility behaviours during the first year following birth may decrease or increase the length of the birth interval [5]. These behaviours include breastfeeding and its influence on return of menses (postpartum amenorrhoea), return to sexual activity (postpartum sexual abstinence) and use of maternal health services. Some of these behaviours biologically reduce fertility while some affect decisions to use family planning during the postpartum period [5].

Women are not at risk of pregnancy following a birth if they are amenorrhoeic as a result of intensive exclusive breastfeeding or abstaining from sex [6]. The period of insusceptibility, which is influenced by sexual abstinence and exclusive breastfeeding, lengthens the time until the next conception. Use of contraception within this period offers extra protection against the risk of pregnancy [7]. The length and intensity of breastfeeding and the length of amenorrhea and sexual abstinence vary among women and within societies. In this respect, postpartum behaviours need to be understood in relation to the contraception behaviours of women [7] if unintended pregnancies are to be avoided. The objectives of this study were to: assess how protected postpartum women in the Mfantseman municipal were against pregnancy, based on their patterns of amenorrhoea and sexual abstinence; determine the timing of postpartum contraception in relation to amenorrhoea and sexual abstinence; and determine the predictors of postpartum contraceptive use.

## Methods

### Study area

The study was carried out in the Mfantseman Municipal area of the Central Region of Ghana. This is a coastal and predominantly rural district in the southern part of Ghana. The main ethnic group is Fante. The main occupation of the people in the district are farming, fishing and trading.

The area was selected because of the high levels of teen pregnancies (13.6% of all pregnancies) high abortion rates amongst the teens and very low family planning uptake which had been consistently below 10% over a three year period from 2007 to 2009 according to the 2010 annual report of the Municipal Health Directorate [8].

### Study design and data source

This was a prospective study of postpartum women who had been encountered earlier at antenatal clinic in four selected health facilities in the Mfantseman Municipal area (Saltpond hospital, Mankessim health centre, Anomabo and Biriwa health centres) where their intention to use postpartum family planning was ascertained. These women agreed to be followed up to their communities within a year following delivery to ascertain their postpartum reproductive and contraceptive behaviours. Data on duration of breastfeeding, amenorrhoea and sexual abstinence, and time of first contraceptive use, continuation or discontinuation, were obtained within one calendar year after last birth using a reproductive event sheet adapted from DHS contraceptive calendar. The data sheets captured month-by-month data on contraceptive use or non-use, continuation or discontinuation of contraception, breastfeeding patterns, sexual behaviour and menstrual resumption to cover period since last birth. The data sheets were reviewed one year postpartum at the time of interview; however, periodic contacts with the respondents were done through telephone calls, home visits and personal contacts to ensure they were capturing data consistently and to clarify any issues that may have arisen. Reasons for use, non-use and discontinuation if any, were obtained from participants at the time of the interview. Information on socio-demographic and socio-economic characteristics was also obtained.

### Sample size estimation and sampling

Based on an estimated target population of 4218 (Average total number of women visiting ANC at all selected health facilities per quarter, per year from 2008 to 2010) and the assumption that 50% of pregnant women intended to adopt postpartum family planning, within a margin of error of 3%, a minimum sample size ST, was estimated as follows: For a finite population, the sample size ST, was estimated by the formula: ST = A / [1 + (A-1)/T] [9], where A is given by [Z^2^*P*(1-P)] / C^2^; T = estimated target population; Z = Z value (1.96 for 95% confidence); P = Proportion of pregnant women who intended to adopt postpartum family planning; and C = margin of error. This implies, A = [1.96^2^ (0.5) (0.5)]/0.03^2^ = 1067 and ST =1067 / [1+ (1067–1)/4218] =852; (approximated to 900). To take care of defaults and late ANC registrations (each constituting 50% of computed sample size) respectively [10], the minimum sample size (ST = 900) was doubled to 1800 with an additional 10% mark-up for women who would decline to be interviewed. The estimated total sample size was 1980. Within the survey period (2nd January to 30th April 2012), each of the selected health facilities was visited during the days designated for antenatal clinic. At the selected health facilities, all antenatal registrants (pregnant women), irrespective of the period of gestation, who lived in the Mfantseman Municipal area and who were aged 15 to 49 years, were targeted to be part of the study. During the prenatal phase of the study, 1914 antenatal registrants were encountered. Sampling of these was by total enumeration technique. Each of the antenatal registrants was asked if they would wish to be followed up a year after they had delivered to find out about contraceptive use and other reproductive health behaviours. Only 71% (1359) of them agreed to be followed up after delivery. The other 29% declined follow up for religious, socio-cultural and personal reasons considering the sensitive nature of family planning within such communities.

Several strategies to further reduce non-response and attrition and improve retention rates were employed in this study. These included the following: first, giving detailed explanations about the study objectives and its possible impacts on the individual, family and society and allaying any anxieties and fears about participating in the study, whilst ensuring that emotional support was on hand to deal with extreme cases; second, obtaining detailed personal information including names, telephone numbers, house addresses (where available) and detailed descriptions of directions to houses of respondents; third, providing adequate motivation for participation by ensuring that the research assistants were friendly, showed respect and courtesy to the respondents and provided adequate privacy at the environment of the interview; fourth, improving rapport between research team and respondents by making periodic contacts with the respondents who agreed to be followed up through telephone calls, home visits and personal contacts; fifth, providing learning opportunities to the research assistants by the comprehensive training given. This motivated the research assistants to engage the respondents in ways that improved rapport and encouraged participation; sixth, providing research assistants in Phase 2 additional incentives for transportation to help them access all the respondents assigned to them, especially to the remotest parts of the municipality and seventh, regular sensitization about the study carried out by the Municipal Health Directorate through their health centres, outreach points and home visits.

These strategies notwithstanding, only 1003 (74%) of the 1359 who agreed to be followed up were encountered. Twenty-six percent (26%) could not be traced mainly because of poor address systems in the communities and non-functional telephone numbers. Follow up interviews were carried out from 2nd January to 30th May 2014. These were done via face-to-face and telephone interviews using home addresses and telephone numbers provided.

### Data management and analysis

The data obtained were double-entered using EPI-DATA, verified and cleaned. The cleaned data were exported into STATA (version 11) for analysis. Descriptive, chi square and logistic regression statistics were carried out to describe the socio-demographic and socio-economic characteristics of respondents, determine associations between contraceptive use and breastfeeding, amenorrhoea and sexual abstinence and determine the predictors of postpartum contraceptive use, respectively. Survival analysis techniques were used to assess the time to resumption of menstrual flow, resumption of sexual activities and time to first contraceptive use.

## Results

### Socio-demographic characteristics of respondents

Table 1 presents the distribution of socio-demographic characteristics of respondents in this study. The mean age of the respondents was 29.9 ± 6.5 years. Majority (73.9%) of the women were in their prime age (20–34 years) whilst adolescents constituted the least proportion (3.3%) of the women. More than half (54.1%) of the women had attained Middle, Junior secondary school (JSS) or Junior high school(JSS) education and a little above 8% (8.3%) had no formal education. The ethnic group with the highest proportion was Fante (88.7%). Christians were the majority (90.3%) religious group among the women, followed by the Muslims (7.6%). Most (43.3%) of the women had married by means of traditional rites and more than half (51.4%) of them were petty traders.

**Table 1:** Socio-demographic Characteristics of Respondents

Variable	Number *N* = 1003	Percentage (%)
Age		
15–19	33	3.3
20–34	741	73.9
35–39	132	13.2
40+	97	9.7
Educational level		
None	83	8.3
Primary	214	21.3
Middle/Junior secondary School/Junior high school	543	54.1
Secondary/Senior secondary school/Senior high School /Vocational	119	11.9
Tertiary	44	4.4
Ethnicity		
Fante	890	88.7
Efutu	18	1.8
Other Akan	46	4.6
Ewe	21	2.1
Hausa	22	2.2
Other	6	0.6
Religion		
Christian	906	90.3
Muslim	76	7.6
Other	21	2.1
Occupation		
Fishmonger	69	6.9
Farmer	33	3.3
Petty Trader	515	51.4
Civil/Public Servant	111	11.1
Student	32	3.2
Other	243	24.2
Area of residence		
Saltpond	316	31.5
Biriwa	135	13.5
Anomabo	179	17.9
Mankessim	259	25.8
Other	114	11.4
Marital Status		
Married through church/mosque/court wedding	213	21.2
Married only by traditional rite	434	43.3
Engaged, yet to be married	240	23.9
Co-habitation (living together)	73	7.3
Divorced/Separated/Widow/Single	43	4.3

Source: Field data (2012 and 2014)

### Breastfeeding, amenorrhoea, sexual abstinence and contraceptive use postpartum

The mean durations of breastfeeding, amenorrhoea and sexual abstinence were 6.6 ± 2.8 months, 7.8 ± 3.8 months and 4.4 ± 3.1 months respectively. The mean time of first contraceptive uptake following the last birth among women who had used contraceptives was 3.5 ± 2.7 months. Table 2 shows the length of breastfeeding, amenorrhoea and sexual abstinence and time of first contraceptive uptake since the last birth. Among those who breastfed, majority (71.6%) had breastfed for six months whilst majority (62.5%) had remained amenorrheic for seven months up to a year.

**Table 2:** Length of breastfeeding, Amenorrhoea and Sexual abstinence and time of first contraceptive use

Months after delivery	Breastfeeding (Yes)		Amenorrhoea (Yes)		Sexual abstinence (Yes)		First PPFP use (Yes)	
	*N* = 860		*N* = 962		*N* = 761		*N* = 422	
	n(%)	cum.%	n(%)	cum.%	n(%)	cum.%	n(%)	cum.%
1	8(0.9)	0.9	40(4.2)	4.2	111(14.6)	14.6	78(18.5)	18.5
2	14(1.6)	2.5	12(1.3)	5.5	128(16.8)	31.4	102(24.2)	42.7
3	7(0.8)	3.3	87(9.0)	14.5	111(14.6)	46.0	96(22.7)	65.4
4	13(1.5)	4.8	50(5.2)	19.7	154(20.2)	66.2	38(9.0)	74.4
5	105(12.2)	17.0	134(13.9)	33.6	40(5.3)	71.5	30(7.1)	81.5
6	616(71.6)	88.6	37(3.9)	37.5	74(9.7)	81.2	28(6.6)	88.1
7–12	97(11.3)	100	602(62.5)	100	143(18.8)	100	50(11.9)	100

Source: field data 2014

**Fig. 1:**
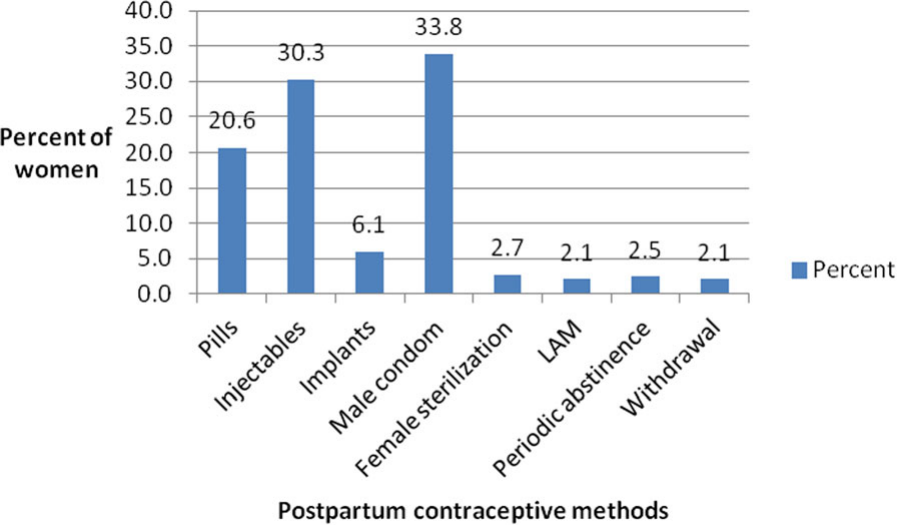
Bar graph showing widely used postpartum contraceptive methods among women in the Mfantseman municipal of the central region of Ghana. Source:Field data 2014. Various postpartum contraceptive methods were used by the women These included oral contraceptive pills (pills), injectables, implants, male condoms, female sterilization, lactational amenorrhoea method (LAM), periodic abstinence and withdrawal method. Of all these methods, the ones that were used by most women were the male condom (33.8%), injectables (30.3%) and the oral contraceptive pills. The least used methods were the withdrawal (2.1%) and lactational amenorrhoea (2.1%) methods

The period of sexual abstinence among the postpartum women was relatively shorter. Majority (66.2%) had abstained for periods of one to four months following birth. Out of the 1003 postpartum women interviewed, 505 (50.3%) of them reported using contraceptives following birth, however only 422 provided correct information regarding time of contraceptive use following birth. None of these women reported any discontinuation once they started using contraceptives of their choices. The 83 women dropped had incorrect or inconsistent data or had missing information on record sheets. Nearly 66% had used contraceptives between the first and third month after birth. The most common methods used were male condoms (33.7%), injectables (30.3%) and pills (20.6%) respectively (Fig. 1).

**Fig. 2:**
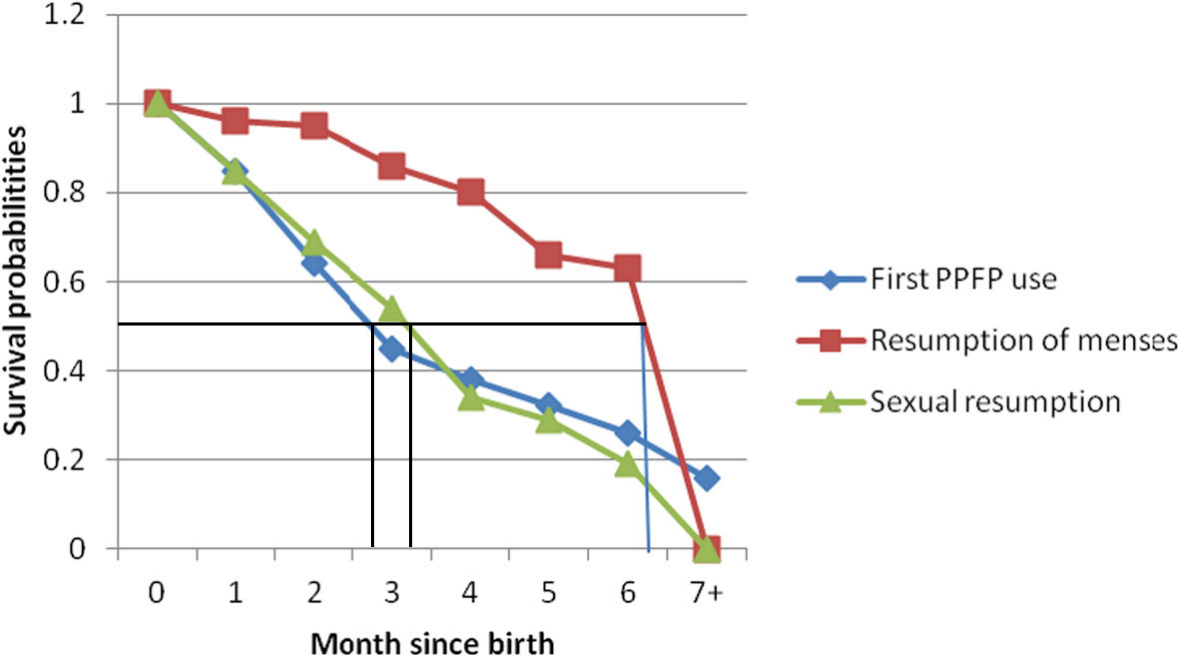
Survival curves depicting first menstrual resumption, sexual resumption and use of postpartum family planning among postpartum women in the Mfantseman municipal. Source: Field data 2014. The X-axis represents the months since delivery, whilst the Y-axis represents the survival probabilities. The red, blue and green line graphs represent the time of resumption of menses, time of postpartum family planning (PPFP) use and time of resumption of sexual following delivery respectively. The figure shows that postpartum family planning use by 50% of the women was at 2.7 months postpartum. This was 0.6 and 3.6 months prior to resumption of sexual relations and menses respectively

**Fig. 3:**
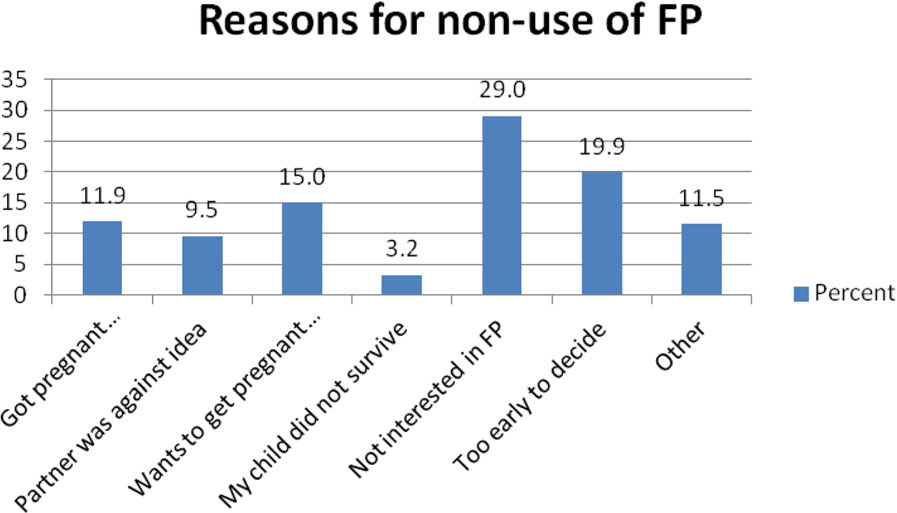
Bar graph showing reasons for non-use of postpartum family planning by women in the Mfantseman municipal. Source: Field data 2014. The women who had not used any family planning methods within the first year after delivery gave several reasons why they had not done so. These included the fact that some got pregnant, some partners were against the idea to do family planning, some women wanted to get pregnant soon, some were not interested in family planning, whilst others thought it was too early to decide. Lack of interest in family planning (29%), too early to take a decision about family planning (19.9%) and women wanting to get pregnant soon (15%) were reasons given by most of the women

Figure 2 shows the survival curves for first sexual resumption, first menstrual resumption and first contraceptive use by ordinal postpartum month. The survival curves show that the time at which 50% of the women had resumed their menses was about 6.3 months postpartum. Also, the time that 50% of them had resumed sex was 3.3 months postpartum, which is 3 months before resumption of first menses. This implies that women who were not using any postpartum contraceptive methods for any reason could be at risk of unintended pregnancy following resumption of sexual relations, especially when the intensity of breastfeeding could not be guaranteed (Reasons for non-use have been provided in Fig. 3). The time to first use of a modern contraceptive method during postpartum period indicates that about 50% of the women had used modern contraceptive methods by 2.7 months postpartum, and occured 0.6 and 3.6 months prior to resumption of sexual relations and menses respectively.

### Predictors of postpartum contraceptive use

Tables 3 and 4 summarize the association between postpartum contraceptive use among women and their socio-demographic and reproductive characteristics respectively. Women’s age (*p* < 0.001), educational level (*p* < 0.001), occupation (*p* < 0.001), area of residence (*p* < 0.001) and marital status (*p* < 0.001) were found to be associated with postpartum contraceptive use. However, ethnicity (*p* = 0.061) and religion (*p* = 0.287) of women were not associated with postpartum contraceptive use. There was an association between postpartum contraceptive use and resumption of sexual relationship (*p* < 0.001), amenorrhoea (*p* < 0.001), and breastfeeding (*p* < 0.001).

**Table 3:** Association between postpartum contraceptive use and socio-demographic characteristics of respondents

Variable	Yes	No	χ^2^	*p*-value
	N=505	N=498		
	n (%)	n (%)		
Age				
15–19	14(42.4)	19(57.6)		
20–34	343(46.3)	398(53.7)		
35–39	87(65.9)	45(34.1)		
40+	61(62.9)	36(37.1)	24.60	< 0.001
Educational level				
None	36(43.4)	47(56.6)		
Primary	102(47.7)	112(52.3)		
Middle/Junior secondary School/Junior high school	306(56.4)	237(43.7)		
Secondary/Senior secondary school/Senior high School	44(37.0)	75(63.0)		
Tertiary	17(38.6)	27(61.4)	21.00	< 0.001
Ethnicity				
Fante	452(50.8)	438(49.2)		
Efutu	3(16.7)	15(83.3)		
Other Akan	22(47.8)	24(52.2)		
Ewe	13(61.9)	8(38.1)		
Hausa	11(50.0)	11(50)		
Other	4(66.7)	2(33.3)	10.11	0.061
Religion				
Christian	459(50.7)	447(49.3)		
Muslim	39(51.3)	37(48.7)		
Other	7(33.3)	14(66.7)	2.50	0.287
Occupation				
Fishmonger	36(52.2)	33(47.8)		
Farmer	7(21.2)	26(78.8)		
Petty Trader	307(59.6)	208(40.4)		
Civil/Public Servant	40(36.0)	71(64.0)		
Student	18(56.3)	14(43.8)		
Other	97(39.9)	146(60.1)	49.09	< 0.001
Area of residence				
Saltpond	157(49.7)	159(50.3)		
Biriwa	60(44.4)	75(55.6)		
Anomabo	130(72.6)	49(27.4)		
Mankessim	107(41.3)	152(58.7)		
Other	51(44.7)	63(55.3)	47.37	< 0.001
Marital Status				
Married through church/mosque/court wedding	129(60.6)	84(39.4)		
Married only by traditional rite	218(50.2)	216(49.8)		
Engaged, yet to be married	94(39.2)	146(60.8)		
Co-habitation (living together)	46(63.0)	27(37.0)		
Divorced/Separated/Widow/Single	18(41.9)	25(58.1)	26.82	< 0.001

**Table 4:** Association between postpartum contraceptive use and reproductive characteristics

Variable	Yes	No	χ^2^	*p*-value
	*N* = 505	*N* = 498		
	n (%)	n (%)		
Outcome of last pregnancy				
Live term baby	498(50.6)	487(49.4)		
Stillbirth/Miscarriage	7(38.9)	11(61.1)	0.96	0.326
Place of delivery				
Home	18(54.6)	15(45.5)		
Public hospital/Private hospital/Maternity home	487(50.2)	483(49.8)	0.24	0.624
Mode of delivery				
SVD	455(53.1)	402(46.9)		
Assisted vaginal delivery	11(13.1)	73(86.9)		
Caesarean section	39(62.9)	23(37.1)	53.12	< 0.001
Delivery complications				
Yes	116(65.9)	60(34.1)		
No	388(47.00)	437(53.0)	20.68	< 0.001
Resumed sexual relationship since last birth				
Yes	471(53.6)	408(46.4)		
No	34(27.4)	90(72.6)	29.76	< 0.001
Ever breastfed after delivery				
Yes	363(46.5)	418(53.5)		
No	142(64.0)	80(36.0)	21.14	< 0.001
Return of menses since last birth (amenorrhoea)?				
Yes	466(52.4)	424(47.6)		
No	39(34.5)	74(65.5)	12.77	< 0.001
	n (Arithmetic mean)	n (Arithmetic mean)		
Date of delivery in months^a^	493(15.7)	490(17.5)		< 0.001
How many Months after birth did you not have your period was not had?^a^	482(8.0)	480(7.6)		0.080
Number of children ever born/Parity of the last birth^a^	505(3.4)	498(2.0)		< 0.001
Preferred number of children^a^	388(3.7)	355(3.2)		< 0.001

^a^Unpaired t-test was used to determine association

Source: Field data (2014

Table 5 presents the univariate and multivariate logistic regression analyses depicting the unadjusted (OR) and adjusted odds ratios (aOR) of factors that influence contraceptive use. All the variables that were associated with postpartum contraceptive use among women (Tables 3 and 4) were used to construct the univariate and the multivariate models.

**Table 5:** Predictors of contraceptive use among postpartum women attending antenatal clinic at Mfantseman Municipal

	Univariate Model		Multivariate Model	
Variable	OR (95% CI)	*p*-value	aOR (95% CI)	*p*-value
Age				
15–19	1		1	
20–34	1.16(0.58–2.37)	0.663	0.47(0.14–1.57)	0.221
35–39	2.62(1.20–5.72)	0.015	0.48(0.12–1.89)	0.292
40+	2.30(1.03–5.13)	0.042	0.47(0.11–2.02)	0.308
Educational level				
None	1		1	
Primary	1.19(0.71–1.98)	0.506	1.86(0.73–4.75)	0.192
Middle/Junior secondary school/Junior high school	1.69(1.06–2.69)	0.028	2.00(0.85–4.71)	0.115
Secondary/Senior secondary school/Senior high school	0.77(0.43–1.35)	0.361	2.22(0.80–6.16)	0.126
Tertiary	0.82(0.39–1.73)	0.607	1.84(0.39–8.62)	0.441
Occupation				
Fishmonger	1		1	
Farmer	0.23(0.09–0.64)	0.004	0.20(0.03–1.28)	0.090
Petty Trader	1.35(0.82–2.24)	0.240	2.23(0.96–5.16)	0.061
Civil/Public Servant	0.52(0.28–0.95)	0.034	1.94(0.57–6.62)	0.288
Student	1.18(0.51–2.74)	0.703	5.00(1.12–22.40)	0.035
Other	0.61(0.36–1.04)	0.071	1.46(0.60–3.53)	0.405
Area of residence				
Saltpond	1		1	
Biriwa	0.81(0.54–1.21)	0.308	2.03(0.90–4.57)	0.089
Anomabo	2.69(1.80–3.99)	< 0.001	1.86(0.98–3.52)	0.059
Mankessim	0.71(0.51–0.99)	0.045	0.58(0.34–0.99)	0.049
Other	0.81(0.53–1.26)	0.365	0.84(0.44–1.60)	0.601
Marital Status				
Married through Church/Mosque/Court wedding	1		1	
Married only by traditional rites	0.66(0.47–0.92)	0.013	1.16(0.68–2.01)	0.581
Engaged, yet to marry	0.42(0.29–0.61)	< 0.001	1.23(0.64–2.41)	0.532
Co-habitation	1.11(0.64–1.92)	0.711	1.47(0.64–3.39)	0.370
Divorced/Separated/Widowed/Single	0.47(0.24–0.91)	0.026	0.27(0.07–1.10)	0.068
Mode of delivery				
Spontaneous Vaginal Delivery	1		1	
Assisted vaginal delivery	0.13(0.07–0.25)	< 0.001	0.20(0.06–0.70)	0.012
Caesarean section	1.50(0.88–2.55)	0.137	5.12(2.24–11.68)	< 0.001
Any delivery complications				
Yes	1		1	
No	0.46(0.33–0.65)	< 0.001	2.14(0.92–4.94)	0.076
Resumed sexual relationship since birth				
Yes	1		1	
No	0.33(0.22–0.50)	< 0.001	0.89(0.41–1.98)	0.784
Ever breastfed after delivery				
Yes	1		1	
No	2.04(1.50–2.780)	< 0.001	2.16(1.11–4.21)	0.024
Return of menses since your last birth				
Yes	1		1	
No	0.48(0.32–0.72)	< 0.001	1.65(0.81–3.38)	0.168
Date of delivery in months	0.90(0.88–0.93)	< 0.001	0.89(0.84–0.95)	< 0.001
Preferred number of children	2.16(1.78–2.63)	< 0.001	3.95(2.77–5.62)	< 0.001
Number of children ever born/Parity of the last child	1.90(1.71–2.10)	< 0.001	2.26(1.83–2.78)	< 0.001

Source: Field data (2014)

In the univariate logistic regression, women within the age group 35–39 years were 2.62 times more likely to use postpartum contraceptive as compared to those within the age group 15–19 years (OR = 2.62, 95% CI: 1.20–5.72, *p* = 0.015). Women with ages 40 years or above also had higher odds of using postpartum contraceptive as compared to adolescents (15–19 years). Women with Middle, Junior secondary school or Junior high school (OR = 1.69, 95% CI: 1.06–2.69, *p* = 0.028) also had increased likelihood of using postpartum contraceptive as compared with women without formal education. Civil or public servants were 48% less likely to use postpartum contraceptive as compared to fish mongers (OR = 0.52, 95% CI: 0.28–0.95, *p* = 0.034). Women living in Anomabo (OR = 2.69, 95% CI: 1.80–3.99, *p* < 0.001) and Mankessim (OR = 0.71, 95% CI: 0.51–0.99, *p* = 0.045) had higher and lower odds respectively of using postpartum contraceptive as compared to women living in Saltpond. Moreover, women who were married through traditional rites (OR = 0.66, 95% CI: 0.47–0.92, *p* = 0.013), engaged (OR = 0.42, 95% CI: 0.29–0.61, *p* < 0.001) and were single, separated, widowed or divorced (OR = 0.47, 95% CI: 0.24–0.91, *p* = 0.026) were less likely to use postpartum contraceptive. Women with assisted vaginal delivery were 87% less likely to use postpartum contraceptive as compared to women who had spontaneous vaginal delivery (SVD) (OR = 0.13, 95% CI: 0.07–0.25, *p* < 0.001). Compared to women who had complications, those who did not have complications had decreased likelihood of using postpartum contraceptive (OR = 0.33, 95% CI: 0.33–0.65, *p* < 0.001). In addition, women who were not breastfeeding had two-fold of using postpartum contraceptive (OR = 2.04, 95% CI: 1.50–2.78, *p* < 0.001). Amenorrhoeic women were 52% less likely to use postpartum contraceptive (OR = 0.48, 95% CI: 0.32–0.72, *p* < 0.001). For a unit increase in the women’s preferred number of children, their odds of using postpartum contraceptive increased more than two-fold (OR = 2.16, 95% CI: 1.78–2.63, *p* < 0.001). For a unit increase in parity, the likelihood of women using postpartum contraceptive increased by 1.9 (OR = 1.90, 95% CI: 1.71–2.10, *p* < 0.001).

### Multivariate regression

In the multivariate logistic regression, occupation (likelihood ratio *p* = 0.013), area of residence (likelihood ratio *p* = 0.004), mode of delivery (likelihood ratio *p* < 0.001), breastfeeding (*p* = 0.024), period since delivery (*p* < 0.001), preferred number of children (*p* < 0.001) and parity (*p* < 0.001) were found to be predictors of postpartum contraceptive use. Women who were students (aOR = 5.00, 95% CI: 1.12–22.40, *p* = 0.035) had higher odds of using postpartum contraceptives. Women living in Mankessim (aOR = 0.58, 95% CI: 0.34–0.99, *p* = 0.049) were less likely to use postpartum contraceptives as compared with those living in Saltpond. Compared with those who had SVD, women who had assisted vaginal delivery were 80% less likely (aOR = 0.20, 95% CI: 0.06–0.70, *p* = 0.012) to use postpartum contraceptive, whereas women who had caesarean section had 5.12 times (aOR = 5.12, 95% CI: 2.24–11.68, *p* = 0.049) likelihood of using postpartum contraceptive. Women who did not breastfeed had more than double likelihood of using postpartum contraceptive (aOR = 2.16, 95% CI: 1.11–4.21, *p* = 0.024) compared to those who breastfed. For a unit increase in the period since delivery, the women were 0.89 times less likely to use postpartum contraceptive (aOR = 0.89, 95% CI: 0.84–0.95, *p* < 0.001). For a unit increase in the preferred number of children, the odds of using postpartum contraceptive increased among women (aOR = 3.95, 95% CI: 2.77–5.62, *p* < 0.001). For a unit increase in parity, the likelihood of women using postpartum contraceptive increased by 2.3 (aOR = 2.26, 95% CI: 1.83–2.78, *p* < 0.001).

## Discussion

The objectives of the study were to: assess how protected postpartum women in the Mfantseman municipal were against pregnancy, based on their patterns of amenorrhoea and sexual abstinence; determine the timing of postpartum contraception in relation to amenorrhoea and sexual abstinence; and determine the predictors of postpartum contraceptive use. The intensity and duration of breastfeeding, length of amenorrhoea and sexual abstinence, individually or in combination affect women’s risk of getting pregnant within the extended postpartum period and before the two-year recommended spacing period after the last birth [1], especially in the absence of contraceptive use. Traditionally, women in sub-Saharan Africa relied mainly on breastfeeding, amenorrhoea and sexual abstinence to space their births. Periods of breastfeeding for as long as two years and its attendant long duration of amenorrhoea in some settings and long periods of sexual abstinence in middle and West Africa, were the norm [11].

Increasing modernization, urbanization and social change have gradually reduced the effectiveness of these traditional birth spacing mechanisms and therefore women have increasingly been at risk of unintended pregnancies in the postpartum period [12]. The mean duration of breastfeeding among the study participants was 6.6 ± 2.8. This is greater than the mean duration of exclusive breastfeeding in Ghana of 3.9 months and less than the mean duration of any breastfeeding of 21.2 months in Ghana and 20.4 months in the central region respectively [13]. The mean duration of any breastfeeding in the municipality was about 14% lower than that of the region and was among the lowest in the region. This may be due to the increase in the use of bottle feeding (which some women consider as modern) which also increased in the country between 2008 and 2014 [13].

Postpartum protection from conception depends upon the intensity and duration of breastfeeding. In the absence of reasonably intensive breastfeeding, women are likely to ovulate before the end of the second postpartum month and hence become susceptible to pregnancy [14]. A woman is considered insusceptible if she is not exposed to the risk of pregnancy, either because she is amenorrhoeic as a result of reasonably intensive breastfeeding exclusively for the first six months or because she is abstaining from sexual intercourse following birth [13]. The median duration of amenorrhoea and sexual abstinence among the respondents were 7.8 and 4.4 months respectively. These were shorter than the median durations of amenorrhoea and sexual abstinence for central region (9.9 and 6.6 months) and Ghana (8.4 and 5.9 months) respectively [13]. The relatively shorter durations of amenorrhoea may be due to the shorter duration and lower intensity of breastfeeding among the women in the municipality. The relatively shorter durations of sexual abstinence and amenorrhoea and the lower intensity of breastfeeding may expose the women in the municipality to the risk of unintended pregnancy, especially in the absence of contraceptive use. This may explain the high rates of unintended pregnancy among pregnant women in the municipality [15].

Among the women who used contraceptives, time of initiation was earlier than initiation of sexual relations and first menses following their last birth. Mean time of initiation of contraceptive was 3.5 months following birth. Among this group of women, 50% had initiated contraceptive use 0.6 and 3.6 months earlier than initiation of sexual relations and first menses respectively at 2.7 months after birth. This was in contrast with what was observed in a study in urban slums in Nairobi where initiation of postpartum contraceptive use by 50% of the women had occurred 7 months after birth, 4 months after initiation of sexual relations and a month after first menses [12]. The relationship observed between postpartum contraceptives use, the initiation of sexual relations and first menses in the Mfantseman municipal is not very common in sub-Saharan Africa [16–18]. The finding that women who adopted contraceptives did so early and prior to resumption of sexual relations and first menses is generally encouraging; however, it must be borne in mind that it does not guarantee the prevention of unintended pregnancies and adequate spacing of births, unless the methods adopted were not discontinued or unless permanent methods were adopted. In this study, the women after initiation of contraceptives did not report discontinuation within the first year postpartum. The most widely used methods adopted among the women in the Mfantseman municipal were male condoms, followed by injectables and pills. In Ghana, the most widely used methods among married women were injectables, implants and pills, whilst male condoms, pills and injectables were the most widely used among unmarried women [14]. The methods used among the women were different because there were several methods available and the women had the freedom to choose which methods were suitable for them. Among postpartum women in a study among urban slum dwellers in Nairobi, the most widely used methods were injectables and pills [12].

It is not easy to predict how early the return of ovulation and menses for a particular woman who did not breastfeed would be. However, evidence exist that women who do not breastfeed after birth usually have early return of ovulation and menses and are thus at greater risk of unintended pregnancies. Education about this fact is provided by midwives during antenatal care in Ghana and other settings. In this study women who did not breastfeed after birth were found to be more likely to use postpartum contraceptives compared to those who breastfed. This is consistent with a study in Egypt where women who exclusively breastfed were less likely to use modern methods of contraception postpartum [19].

In this study, women who underwent caesarean section were five times likely to use postpartum contraceptives compared to those who delivered via spontaneous vaginal delivery. In contrast, those who had assisted vaginal delivery were less likely to use postpartum contraceptives. The reasons for this difference are not very clear in this study setting; however it is known that women who underwent caesarean section were often inclined to use some form of postpartum contraception and preferred methods like bilateral tubal ligation, intrauterine device and injectables [20, 21] in order to avoid repeat caesarean section from another pregnancy too soon. Consistent with a study in Iran [22], place of residence influences contraceptive use among postpartum. Women living in Mankessim were less likely to use postpartum contraceptives compared to those resident in Saltpond. This was unexpected in the sense that women from Mankessim had the highest awareness (83%) about postpartum contraceptives [15] yet were the least likely to use them. Myths, fear of side effects and opposition by significant others may likely influence this observation. Further inquiry may be required to ascertain these facts. The finding in this study that students were more likely than fishmongers to adopt postpartum contraceptives is encouraging in the sense that girls may need to go back to school to complete and advance their educational status in line with the Ghana Education Service’s policy and also to avoid any future unintended pregnancies. High Parity has been found in several studies to be associated with contraceptive use [23–25]. The finding in this study is similar. Women of high parity may have satisfied the number and sex of their children and may wish to limit their births.

### Strengths and limitations of study

The prospective design was appropriate for the study considering the fact that accurate timing of postpartum reproductive health events was important to establish the relationship between them and contraceptive use. Although some strategies were put in place to minimize non-response and attrition (see methodology), the rate nevertheless was still very high (wave I response rate of 97% versus wave II response rate of 51%) and lead to sampling bias, reduced generalizability of study results and increased variance of study estimates. The threat of selection bias existed, but was highly mitigated by ensuring that the data collectors explained the study objectives and their implications very well to the respondents. The study findings may not be generalizable to the national and regional populations but to coastal and predominantly rural populations within the central region and country. The absence of data on duration of family planning use, seriously limits any conclusions about pregnancy risk in the first postpartum year. Some of the data collectors abandoned the study because of inadequate remuneration. New data collectors had to be trained to continue data collection. This brought about some delays in data analysis and reporting.

## Conclusion

The objectives of this study were to assess how protected postpartum women in the Mfantseman municipal were against pregnancy, based on their patterns of amenorrhoea and sexual abstinence; determine the timing of postpartum contraception in relation to amenorrhoea and sexual abstinence; and determine the predictors of postpartum contraceptive use. Postpartum women in the Mfantseman municipal who did not use contraceptives or delayed in the use of contraceptives after birth were least likely to be protected against pregnancy in the post partum period because of early initiation of postpartum sexual relations, poor intensity and relatively shorter duration of breastfeeding. Women who adopted postpartum family planning were likely to be better protected because they were likely to adopt it within the first three months after birth and before the onset of sexual relations and first menses. The predictors of postpartum contraceptive use were breastfeeding pattern, occupation, parity, preferred number of children, place of residence and mode of delivery.

## Abbreviations

FP: Family Planning
GSS: Ghana Statistical Service
PPFP: Postpartum family planning
SVD: Spontaneous vaginal dellivery
WHO: World Health Orgaization
